# Interspecific Small Molecule Interactions between Clinical Isolates of *Pseudomonas aeruginosa* and *Staphylococcus aureus* from Adult Cystic Fibrosis Patients

**DOI:** 10.1371/journal.pone.0086705

**Published:** 2014-01-23

**Authors:** Alexandre Fugère, David Lalonde Séguin, Gabriel Mitchell, Eric Déziel, Valérie Dekimpe, André M. Cantin, Eric Frost, François Malouin

**Affiliations:** 1 Centre d′Étude et de Valorisation de la Diversité Microbienne (CEVDM), Département de biologie, Faculté des sciences, Université de Sherbrooke, Sherbrooke, Quebec, Canada; 2 INRS-Institut Armand-Frappier, Laval, Quebec, Canada; 3 Service de pneumologie, Département de médecine, Faculté de médecine et des sciences de la santé, Université de Sherbrooke, Sherbrooke, Quebec, Canada; 4 Département de microbiologie et d′infectiologie, Faculté de médecine et des sciences de la santé, Université de Sherbrooke, Sherbrooke, Quebec, Canada; Queens University Belfast, Ireland

## Abstract

*Pseudomonas aeruginosa* and *Staphylococcus aureus* are the most prevalent pathogens in airway infections of cystic fibrosis (CF) patients. We studied how these pathogens coexist and interact with each other. Clinical isolates of both species were retrieved from adult CF patients. Culture supernatants from 63 *P. aeruginosa* isolates triggered a wide range of biofilm-stimulatory activities when added to the culture of a control *S. aureus* strain. The extent of biofilm formation by *S. aureus* was positively correlated to the levels of the 2-alkyl-4-(1*H*)-quinolones (AQs) *Pseudomonas* Quinolone Signal (PQS) and 2-heptyl-4-hydroxy quinoline *N*-oxide (HQNO) produced by the *P. aeruginosa* isolates. Supernatants from *P. aeruginosa* isogenic mutants deficient in PQS and HQNO production stimulated significantly less biofilm formation by *S. aureus* than that seen with the parental strain PA14. When studying co-isolated pairs of *P. aeruginosa* and *S. aureus* retrieved from patients showing both pathogens, *P. aeruginosa* supernatants stimulated less biofilm production by the *S. aureus* counterparts compared to that observed using the control *S. aureus* strain. Accordingly, some *P. aeruginosa* isolates produced low levels of exoproducts and also some of the clinical *S. aureus* isolates were not stimulated by their co-isolates or by PA14 despite adequate production of HQNO. This suggests that colonization of the CF lungs promotes some type of strain selection, or that co-existence requires specific adaptations by either or both pathogens. Results provide insights on bacterial interactions in CF.

## Introduction

Cystic fibrosis is a common life-shortening autosomal recessive disorder that manifests as clinical syndromes of chronic pulmonary infections, gastrointestinal disorder, nutritional and other abnormalities. However, the majority of individuals with CF ultimately succumb to respiratory failure primarily caused by chronic airway bacterial infections from mixed microbial communities [1]. In many cases, *Staphylococcus aureus* is predominant in infections occurring during childhood but colonization by *Pseudomonas aeruginosa* gradually increases as CF patients grow older. Nevertheless, *S. aureus* persists and is one of the most prevalent pathogens in adult CF patients along with *P. aeruginosa* [2], [3], [4]. Despite such documented and frequent co-colonization of *S. aureus* and *P. aeruginosa*, very little is known about the impact of polymicrobial infections on patient health. Also, it is now becoming obvious that bacteria found in CF airways interact together in several different ways [5], [6], [7], [8], [9], [10], [11], [12]. It has been proposed that *S. aureus* could prepare the respiratory tract epithelia of CF patients to promote the subsequent colonization of *P. aeruginosa* [13]. Studies are needed to understand how these microbial interactions develop, affect the balance of colonization and the progression of disease [14].

*P. aeruginosa* produces many extracellular molecules during its different growth phases. Synthesis of many of these compounds is controlled by quorum sensing (QS) regulatory systems. Their impact on other bacterial populations has also become an important research topic. Among QS-controlled pseudomonal factors, LasA, pyocyanin, hydrogen cyanide, and alkyl quinoline *N*-oxides have been reported to reduce *S. aureus* growth [15], [16], [17]. Prolonged growth of *S. aureus* with physiological concentrations of the *P. aeruginosa* exoproduct 4-hydroxy-2-heptylquinoline-*N*-oxide (HQNO) induces a sub-population of slow-growing, respiratory deficient *S. aureus* named small-colony variants (SCVs) [16]. We have also shown that *S. aureus* biofilm production is increased in presence of *P. aeruginosa* supernatant or HQNO, and that the staphylococcal stress- and colonization-related alternative sigma factor B (SigB) is involved in this response [18]. Another class of pseudomonal QS-regulated molecules, the *N*-acylhomoserine lactones (AHLs), was also reported to affect *S. aureus* populations. At sub-inhibitory concentrations, long chain AHLs like *N*-(3-oxododecanoyl)-L-homoserine lactone reduce exotoxins production and down-regulate both *agr* and *sarA* expression in *S. aureus* [7]. To add to the complexity of these bacterial interactions, previous studies have demonstrated that the formation of biofilm by *S. aureus* can be influenced by *agr* [19], *sigB* [20] and *sarA* [21], while *P. aeruginosa* also produces the QS-regulated biosurfactant rhamnolipids, which can disrupt biofilms and show some inhibitory activity on Gram positive bacteria, such as *S. aureus*. [22], [23], [24].

Biofilms may play a major role in the development of chronic lung infections in CF. They confer to bacteria protection from host defenses as well as tolerance to some antibiotics that target active and dividing cells [13], [25]. In addition, biofilms also provide protection against hydrodynamic shear forces, and biofilm infections are generally difficult to eradicate even in hosts with intact immune system [26], [27]. In fact, it was estimated that biofilm formation is involved in 65% of nosocomial infections in the United States [28] and most chronic infections [29] [30]. Naturally-occurring biofilms represent complex and integrated polymicrobial communities in which cell-to-cell signals may alter bacterial growth and virulence [31], [32].

Considering the genetic diversity and hypermutability of *P. aeruginosa* isolates from CF lungs [33], [34], [35], the susceptibility of *S. aureus* to various *Pseudomonas* exoproducts and the complex regulatory mechanisms involved in *S. aureus* biofilm production, a variety of patient-specific interactions between *P. aeruginosa* and *S. aureus* is likely to exist. The aim of this study was to investigate the types and consequences of the interactions between *S. aureus* and *P. aeruginosa* co-isolated from adult CF patients. Measurements of some relevant exoproducts were done to demonstrate the complexity and heterogeneity of *P. aeruginosa* isolates and to determine the impact of such molecules on *S. aureus* biofilm formation, a relevant virulence determinant.

## Materials and Methods

### Ethics statement

This study was approved by the ethics review board of the CHUS (Comité d′éthique de la recherche en santé chez l′humain du Centre Hospitalier Universitaire de Sherbrooke) under Protocol 06-158-R6. Informed written consent was obtained from all subjects (all adults over 18 years of age) prior to recruitment to the study; a document explaining the study objectives and procedures was read to the subject and two copies were signed, one for the subject and the other for our files and for review by the ethic committee. This consent procedure was also reviewed and approved by the ethic committee prior to the study. An annual review and re-approval process was also requested by the ethic committee. Approval was obtained for each of the three years of the study.

### Bacterial strains, media and growth conditions

*P. aeruginosa* PA14 was the prototypical reference strain used in the present study [36]. The *P. aeruginosa* PA14-derived mutants *pqsA, pqsE, pqsH* and *pqsL* were all previously described [37], [38], [39], as were the *lasR/rhlR* double-mutant and the *rhlA* mutant [40], [41]. The relevant properties of these mutants are listed in Table 1. *S. aureus* CF1A-L is a strain isolated from a CF patient and its characteristics in relation to biofilm production and response to some *P. aeruginosa* PA14 exoproducts have already been described [18]. *S. aureus* CF1A-L was thus used as a control strain is this study. For routine maintenance, *P. aeruginosa* was grown on TSA (BD, Mississauga, ON, Canada) and subcultured in TSB (BD), whereas *S. aureus* was grown on BHI agar and broth (BD, Mississauga, ON, Canada).

**Table 1:** *P aeruginosa* reference and mutant strains.

Strain	Relevant characteristics	Consequence or principal compound for which biosynthesis is altered	Reference
*P. aeruginosa*			
PA14	Clinical isolate UCBPP-PA14, Rif^R^	None	[36]
PA14Δ*lasR/rhlR*	PA14 *lasR/rhlR*; Gm, Tc	Altered in QS circuitry and all AQs production	[40]
PA14Δ*pqsA*	PA14 *pqsA*::TnphoA; Rif^R^, Km^R^	HHQ; PQS*, HQNO*	[37]
PA14Δ*pqsE*	PA14 *pqsE*; Amp^R^	Altered in QS circuitry and required for pyocyanin production	[37]
PA14Δ*pqsL*	PA14 Δ*pqsL*; Rif^R^	HQNO	[38]
PA14Δ*pqsH*	PA14 *pqsH*::*aacC1* cassette; Gm^R^, Km^R^	PQS	[39]
PA14Δ*rhlA*	PA14 *rhlA*::TnMrT7;	Rhamnolipids	[41]

* Altered biosynthesis of the precursor molecule.

### Clinical isolates

Clinical isolates of *P. aeruginosa* and *S. aureus* were obtained from 32 adult CF patients at the CF outpatient clinic of the *Centre Hospitalier Universitaire de Sherbrooke* (CHUS, Quebec, Canada). The age of patients ranged from 18 to 41 year old (mean of 25). Through routine visits or during hospitalization (exacerbation), sputum samples were obtained by expectoration or by deep throat swab if no bronchial secretions were produced by the patient. Up to 6 samples were obtained for some patients over a period of 3 years. For all specimens, detection of *S. aureus* and *P. aeruginosa* was made by routine methods. Specimens were streaked on sheep blood agar, MacConkey and mannitol salt agar (MSA) and incubated at 35°C for a minimum of 48 hours. Suspected *P. aeruginosa* colonies growing on MacConkey were confirmed by the oxidase test and their capability to grow at 42°C, and by the Vitek II XL AutoMicrobic System Gram-negative identification card (bioMerieux Vitek, Hazelwood, Mo.). To facilitate detection and confirm the identification of *S. aureus*, a portion of each specimen was also treated as follows. For sputum samples, 100 µl of DTT (100 mg/ml) were added to 100 mg of sputum [42]. From this solution, 100 µl were then added to 10 ml of NaCl broth that consisted of 7.5 g tryptone, 1.25 g neutralized liver digest, 2.5 g yeast extract, 37.5 g NaCl, 5 g D-mannitol, and 2 g of glucose in a final volume of 500 ml. For deep throat specimens, swabs were directly used to inoculate 10 ml of NaCl broth. Cultures were aerobically incubated at 35°C with shaking (225 rpm) for 48 h. Aliquots were then plated on MSA for detection and isolation of *S. aureus*. Suspected *S. aureus* colonies were confirmed with the Pasteurex Staph Plus test (Bio-Rad Laboratories, Marnes-La-Coquette, France), detection of catalase and by PCR detection of the *femA* gene [43] and of the 668 pb product amplified from the *nuc* gene by using the following primer sequences: *nuc* FWD 5′-GGCATCTAGAGCTAAGTCGTGGCATATGTATGGC-3′ and *nuc* REV 5′-CCGCACTAGTCCTTGACCTGAATCAGCGTTG-3′. The *mecA* gene coding for the low-affinity penicillin-binding protein responsible for methicillin resistance in *S. aureus* [44] was also tested by PCR. All isolates were kept at −86°C until used.

A total of 63 clinical *P. aeruginosa* isolates were selected for further studies and these included 23 that were co-isolated with a *S. aureus* strain. This collection was completed with 21 *S. aureus* strains that were not co-isolated with *P. aeruginosa*. The selected bacterial strains were from different patients or from a sample of the same patient collected at a different visit, which occurred 3–8 months apart. A few strains were from the same sample. The *P. aeruginosa* strains collected from the same patient were screened for mucoidy and pigmentation characteristics and only those with obvious phenotypic differences were considered in the study. *S. aureus* strains collected from the same patient were selected based on their distinctive antibiotic resistance profile or VNTR type (multiple-locus variable-number of tandem repeats, [45]).

### Collection of clinical data

Clinical data were associated with each specimen and included age, sex, medical condition and FEV1 (forced expired volume in 1 second) at the time of the visit. The FEV1 was evaluated by the CF clinic's respirologist and recorded with a Jaeger MasterScope spirometer (VIASYS Healthcare GmbH, Hochberg, Germany). The FEV1 was expressed as a percentage of the predicted value (100%) which depends on the sex, age and body height of each patient. This study was approved by the ethics review board of the CHUS. Informed consent was obtained from all subjects prior to recruitment to the study.

### *P. aeruginosa* culture supernatant preparation

Overnight primary cultures of *P. aeruginosa* were used to inoculate 50 ml of TSB in 125-ml flasks at a dilution of 1:100. Cultures were grown aerobically under agitation (225 rpm) for 20 h at 35°C. Supernatants were retrieved by centrifugation at 2,300×*g* and filter-sterilized using 0.22 µm pore size (VWR, Mont-Royal, Canada) and used immediately. A culture supernatant from *Escherichia coli* K12 grown in TSB was used as a control.

### Pyocyanin extraction and quantification

Four ml of chloroform was added to 5 ml of *P. aeruginosa* culture supernatants prepared as described above. After vigorous vortexing, solutions were let to rest until two distinct phases were apparent. The organic phase contains the pyocyanin and is usually colored (blue-green). The top aqueous phase was discarded without disrupting the organic phase. Pyocyanin was then extracted by adding 1 ml HCl 0.2 M combined to another cycle of vortex and resting of the mixture. The pyocyanin-containing phase then appeared in shades of pink and was retrieved. Aliquots of 200 µl were serially diluted in 96-well plates with sterile phosphate-buffered saline (PBS, Sigma) and absorbance was read at 520 nm, using an Epoch plate reader (BioTek Instruments, Winooski, USA).

### 2-alkyl-4-(1*H*)-quinolones (AQs) and *N*-acyl-homoserines lactones (AHL) extractions

Sixteen µl of an internal standard containing 8 ppm of 5,6,7,8-tetradeutero-3,4-dihydroxy-2-heptylquinoline (PQS-d4), and 4 ppm of 5,6,7,8-tetradeutero-4-hydroxy-2-heptylquinoline (HHQ-d4) were added directly to 2 ml of culture supernatant. After mixing by inversion, a quick spin was done to breakup emulsions and extraction was performed twice with 2 ml of ethyl acetate. The organic phases were combined, evaporated and the residue suspended with 500 µl acetonitrile directly in HPLC vials. Samples were stored at −20°C until analyzed.

### LC/MS analyses

Analyses were achieved with a Micromass Quattro II triple quadrupole mass spectrometer (Micromass Canada, Pointe-Claire, Canada) in positive electrospray ionization mode, interfaced to an HP1100 HPLC equipped with a 4.5×150 mm reverse-phase C_8_ column, as reported [46], [47].

### Biofilm formation

To evaluate the effect of *P. aeruginosa* supernatants on biofilm formation by *S. aureus* CF1A-L (control) or other clinical isolates, the supernatants were filter sterilized and diluted in TSB at concentrations ranging between 0 and 25% (v/v). The diluted supernatants (100 µl) were distributed in a 96-well tissue culture treated flat-bottom polystyrene microtiter plate (BD). A *S. aureus* suspension (0.5 McFarland standard) was then prepared in BHI-0.5% glucose and 100 µl were added to each well of the plate already containing the *P. aeruginosa* supernatant dilutions. The plates were incubated for 48 h at 35°C. The cultures were then discarded and the wells were carefully washed three times with 200 µl PBS. Once dried, the plates were treated for 30 min with 200 µl of a 0.1% (p/v) crystal violet solution (sigma HT90132) to stain the biofilm, rinsed twice with 200 µl water to remove excess dye, and then allowed to dry. Finally, the wells were filled with 200 µl 95% ethanol and incubated at room temperature for 1 h with intermittent agitation. The absorbance of each well at 560 nm was then measured. The assays were repeated three times and included three replicates for each condition tested. For each supernatant concentration tested, dose-dependent results were presented relatively to the normal biofilm production level of *S. aureus* measured in the absence of *P. aeruginosa* supernatant. Also, net biofilm increases were calculated by summation of the differences between biofilm formation quantified at each supernatant concentration and the normal biofilm formation level for each strain.

### Statistics

Statistical analyses were carried out with the GraphPad Prism Software (v.5.00). Tests used for the analysis of each experiment are specified in the figure legends.

## Results

### Clinical isolates of *P. aeruginosa* trigger a wide range of biofilm formation responses in *S. aureus*

**Figure 1:**
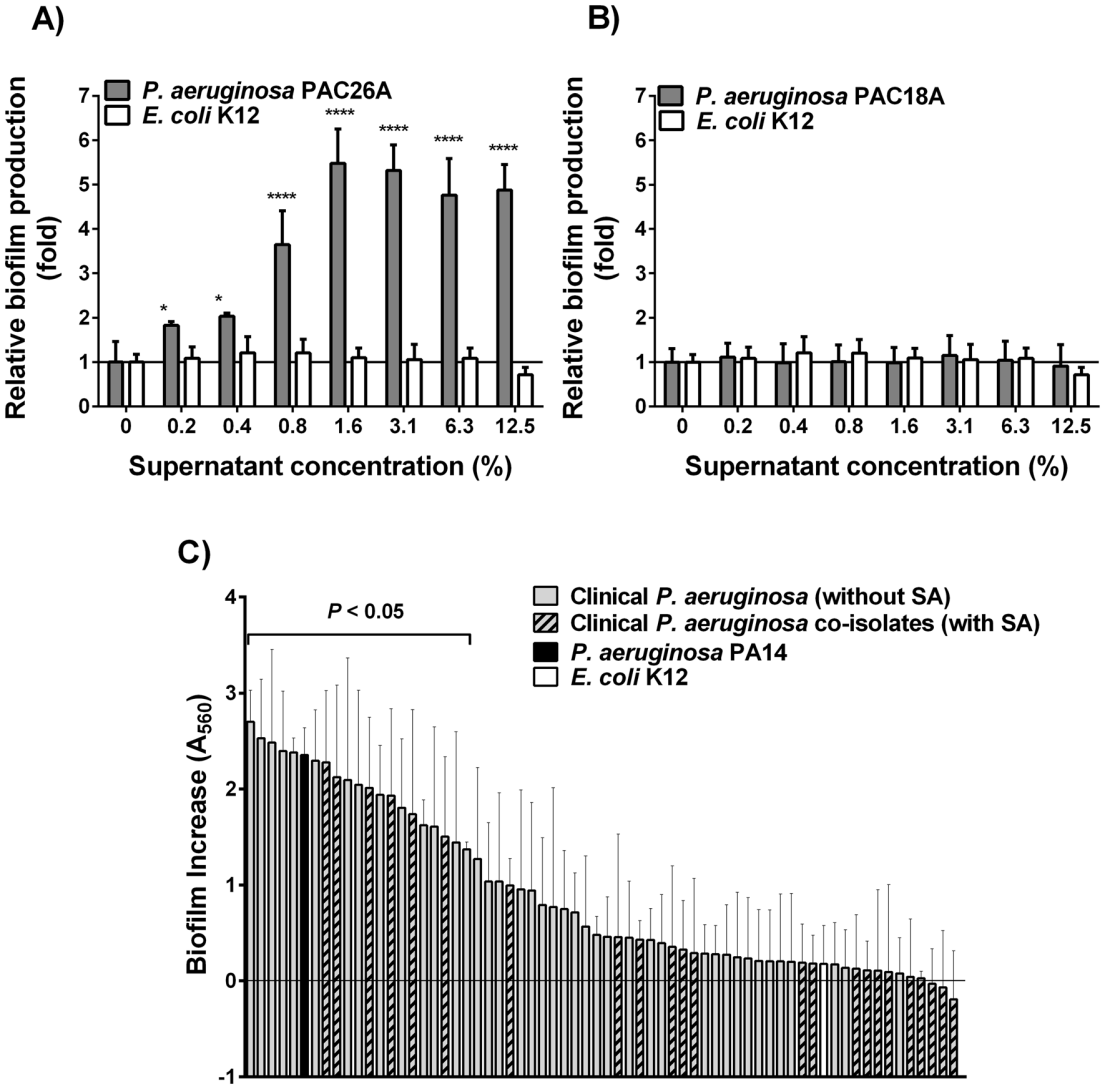
*S. aureus* biofilm stimulation by clinical *P. aeruginosa* supernatants. *S. aureus* CF1A-L biofilm production in response to culture supernatants from clinical isolates of *P. aeruginosa*. (A) Supernatant from the clinical strain *P. aeruginosa* PAC26A stimulates *S. aureus* CF1A-L biofilm formation in a dose-dependent manner, while in (B) a supernatant retrieved from *P. aeruginosa* strain PAC18A does not. Biofilm increase was measured at each supernatant concentration after 48 h of incubation and results were normalized relatively to the biofilm production measured in the absence of *P. aeruginosa* supernatant. Means and standard deviations for triplicates of each supernatant concentration are shown. Statistical significance was determined by a two-way ANOVA and the Bonferroni's multiple comparison post-test (*, *Ρ*<0.05; ****, *Ρ*<0.0001). (C) The dose-dependent biofilm stimulation of *S. aureus* CF1A-L was measured after treatment with supernatants obtained from 63 clinical isolates of *P. aeruginosa*. The mean A_560 nm_ value measured for *S. aureus* CF1A-L biofilm formed in the absence of *P. aeruginosa* supernatant (control condition, 0% supernatant) was subtracted from the A_560 nm_ value detected at each concentration of supernatants (0.2 to 12.5% supernatants as in panels A and B) and summed up in order to calculate the net biofilm increase induced by each *P. aeruginosa* isolate (panel C). Supernatants from *P. aeruginosa* PA14 and *E. coli* K12 were used as positive and negative controls, respectively. Significant differences with the control condition were determined by Two-way ANOVA followed by Sidak's multiple comparisons test.

To measure the effect of *P. aeruginosa* culture supernatants on *S. aureus* biofilm formation, the previously described clinical *S. aureus* strain CF1A-L [18], was used as a reference. This *S. aureus* strain was considered an adequate candidate to act as a control since it shows an increased biofilm production in response to *P. aeruginosa* PA14 supernatant and because it was originally retrieved from a CF patient who was not co-colonized with *P. aeruginosa* [18]. The supernatant from *P. aeruginosa* strain PA14 was used as a positive control for typical production of QS-related compounds [36], [37]. As a matter of fact, the supernatant from a culture of *P. aeruginosa* PA14 stimulates *S. aureus* CF1A-L biofilm formation in a dose-dependent manner with an overall ∼2.5-fold increase in biofilm production compared to that observed in the absence of the *P. aeruginosa* supernatant (*Ρ*<0.001). As expected, biofilm formation was not significantly stimulated in the presence of an *E. coli* K12 supernatant as it does not produce any known stimulatory molecule for *S. aureus* (Fig. S1). Dose-dependent biofilm stimulation was then assayed with supernatants from all 63 clinical isolates of *P. aeruginosa*. Some isolates demonstrated high stimulating ability such as *P. aeruginosa* PAC26A (Fig. 1A) while others acted as poor biofilm inducers like PAC18A, which exhibited no significant biofilm stimulation activity in comparison to the *E. coli* control supernatant (Fig. 1B). A_560 nm_ values measured for crystal violet-stained biofilms formed in the absence of supernatant (control condition, 0% supernatant) were subtracted from the A_560 nm_ values detected at each concentration of supernatant (0.2 to 12.5% *P. aeruginosa* supernatant) and summed up in order to calculate the net biofilm increase induced by each *P. aeruginosa* isolate supernatants. Results revealed a wide distribution of biofilm stimulation activities by the 63 *P. aeruginosa* clinical isolates, among which 30% (19/63) of isolates showed significant stimulation of CF1A-L biofilm production, as calculated with Sidak's multiple comparisons test (Fig. 1C). *S. aureus* CF1A-L biofilm modulation ranged from slight inhibition (−0.19 A_560 nm_) to strong induction (+2.70 A_560 nm_), depending on the *P. aeruginosa* isolate (Fig. 1C).

### *S. aureus* biofilm stimulation by *P. aeruginosa* is associated with PQS and HQNO production

**Figure 2:**
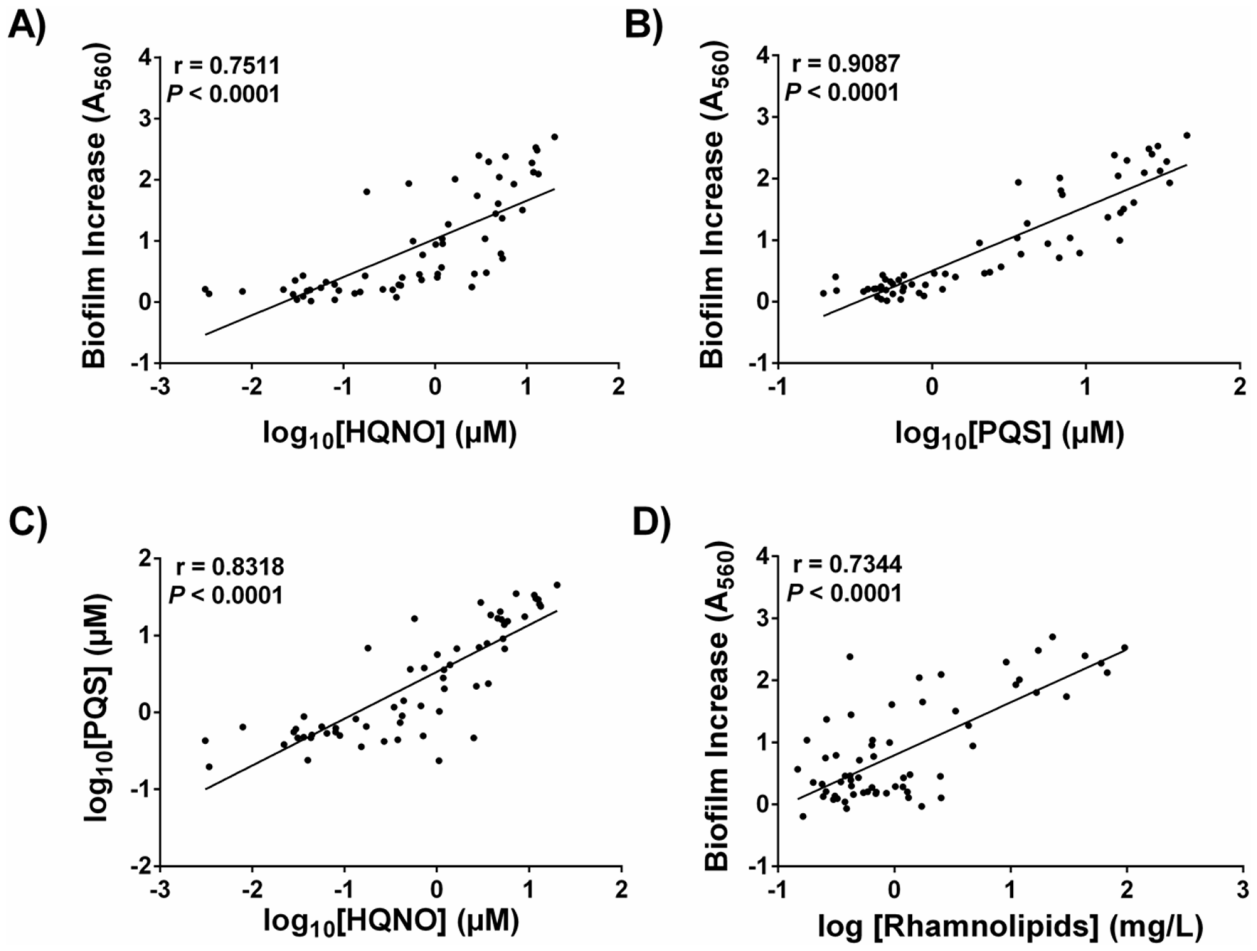
*P. aeruginosa* exoproducts associated with *S. aureus* biofilm stimulation. The ability of *P. aeruginosa* supernatants to stimulate *S. aureus* CF1A-L biofilm formation is correlated with the concentrations of HQNO and PQS. LC/MS was used to detect and quantify specific exoproducts in 20-h culture supernatants of clinical isolates of *P. aeruginosa*. The increase of *S. aureus* biofilm production by *P. aeruginosa* supernatants was calculated as described in Fig. 1C and plotted against the Log10 concentrations of (A) HQNO and (B) PQS found in each supernatant. Pearson's correlation coefficients are indicated and statistical significance was determined by two-tailed *P* value. (C) Correlation between HQNO and PQS production by *P. aeruginosa* isolates using Pearson's calculation. (D) Rhamnolipids amounts produced by *P. aeruginosa* isolates are highly correlated with CF1A-L biofilm increase (Pearson's correlation, two-tailed *P* value).

LC/MS analysis of *P. aeruginosa* supernatants was performed for each clinical isolates in order to determine the concentration of 2-heptyl-4-hydroxyquinoline (HHQ), PQS, HQNO, and AHLs produced after 20 h of growth. Pyocyanin production in these cultures was also measured, as described in the Materials and Methods section. The stimulation of *S. aureus* biofilm formation was found significantly associated with the concentrations of HQNO (r = 0.7511, *P*<0.0001) and PQS (r = 0.9087, *P*<0.0001) in *P. aeruginosa* supernatants, according to Pearson's correlation coefficients (Fig. 2A and 2B). Also, we confirmed that the production levels of HQNO and PQS by *P. aeruginosa* isolates are closely correlated (Fig. 2C, r = 0.8318, *P*<0.0001). As expected, conditions in which biofilm was significantly increased, planctonic bacterial growth was greatly attenuated (data not shown). On the other hand, there was a poor correlation between the concentration of pyocyanin, HHQ or AHLs found in a supernatant and its ability to promote biofilm production by *S. aureus* (Fig. S2). AHLs were not detected in an important proportion of the supernatants analyzed, as C4-HSL was quantified in 58% of the cultures whereas the presence of 3-oxo-C12-HSL was only observed in 19% of the *P. aeruginosa* isolates. (Table S1). Interestingly, the level of rhamnolipids measured in the *P. aeruginosa* supernatants showed a high positive correlation with the capacity to increase biofilm in strain CF1A-L (Fig. 2D, r = 0.7344, *P*<0.0001).

**Figure 3:**
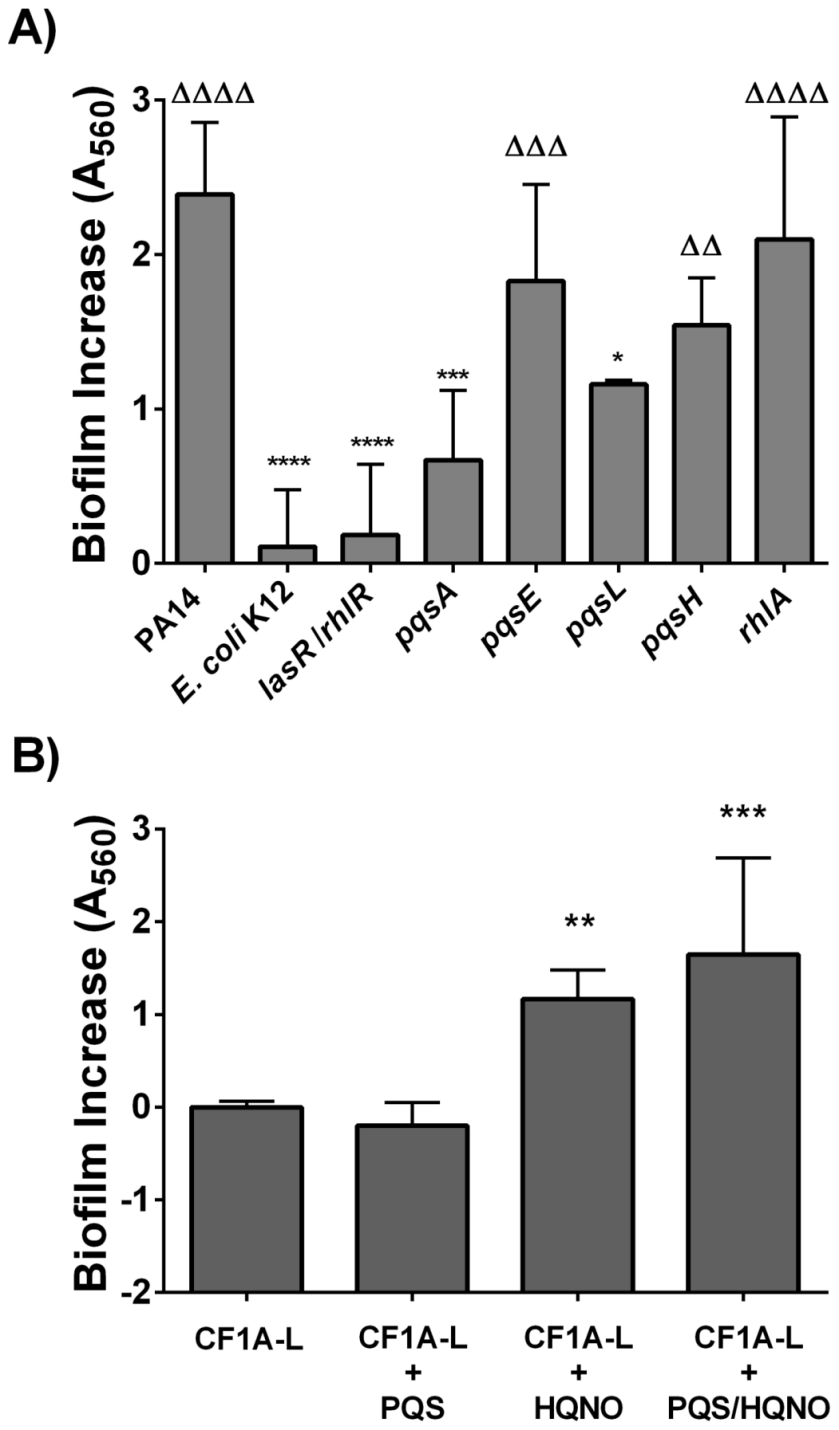
HQNO confirmed as main component in *S. aureus* biofilm stimulation. (A) Stimulation of *S. aureus* CF1A-L biofilm production in response to culture supernatants of *P. aeruginosa* PA14 and isogenic mutants. The supernatant of *E. coli* K12 was used as negative control. Data are presented as means with standard deviations for at least three independent experiments. Statistical significance of results are presented in comparison to those obtained with strain PA14 (*, *P*<0.05; ***, *P*<0.001;****, *P*<0.0001) and *E. coli* K12 (ΔΔ, *P*<0.01; ΔΔΔ, *P*<0.001; ΔΔΔΔ, *P*<0.0001) as calculated by a one-way ANOVA and Bonferroni's multiple comparison test. (B) Biofilm increase (means of total A_560 nm_ variations) measured at different concentrations (0.2, 0.4, 0.8, 1.6, 3.13, 6.25, 12.5, 25.0, 50.0 and 100 µg/ml) of either PQS, HQNO or a PQS/HQNO mixture (1:1) compared to that measured for *S. aureus* CF1A-L grown without supplement. Significant biofilm increases in each condition are indicated (**, *Ρ*<0.01; ***, *Ρ*<0.001) as calculated by one-way ANOVA followed by Dunnett's multiple comparison test.

Isogenic mutants of strain *P. aeruginosa* PA14 altered in the biosynthesis of AQs (Table 1) were then used to confirm or complement our observations. As mentioned above, the culture supernatant of PA14 elicited a great stimulation of *S. aureus* CF1A-L biofilm formation that was statistically significant in comparison to the *E. coli* negative control (*P*<0.0001; Fig. 3A). A significant loss of biofilm induction capacity was observed with the supernatants of the *lasR/rhlR* mutant, altered in the biosynthesis of AHLs and the AQ molecules PQS and HQNO (*Ρ*<0.0001), and of the *pqsA* mutant, deficient in all AQ production (*Ρ*<0.001) (Fig. 3A). This was also the case for the *pqsL* mutant showing a significant reduction in its biofilm stimulation ability (*P*<0.05), thus confirming that HQNO is of utmost importance in the biofilm stimulation process. In addition to the observed correlation between the amount of PQS or HQNO produced by *P. aeruginosa* strains and the biofilm stimulatory activity (Fig. 2), results with the *pqsH* mutant also implies some implication of PQS in biofilm stimulation since this mutant showed a slight decrease in its biofilm stimulatory effect compared to the prototypical PA14 strain (Fig. 3A). Experiments conducted with synthetic PQS (Sigma) showed supporting results since this compound alone did not show any significant effect on *S. aureus* CF1A-L biofilm formation but in combination with HQNO, greatly increased biofilm production to levels appearing higher than those seen with HQNO (Fig. 3B). The latter results act as a chemical confirmation of the specific role of HQNO in the stimulation of *S. aureus* biofilm formation by *P. aeruginosa*.

Besides, our results do not suggest that the presence of the regulator protein PqsE or pyocyanin is linked to *S. aureus* biofilm stimulation since the *pqsE* mutant was not altered in its biofilm stimulation ability (Fig. 3A). Similarly, the *rhlA* mutant induced similar *S. aureus* biofilm stimulation than that observed with PA14, suggesting that rhamnolipids are also not implicated in this phenomenon (Fig. 3A).

### *P. aeruginosa* strains do not stimulate biofilm formation of co-isolated *S. aureus* strains

**Figure 4:**
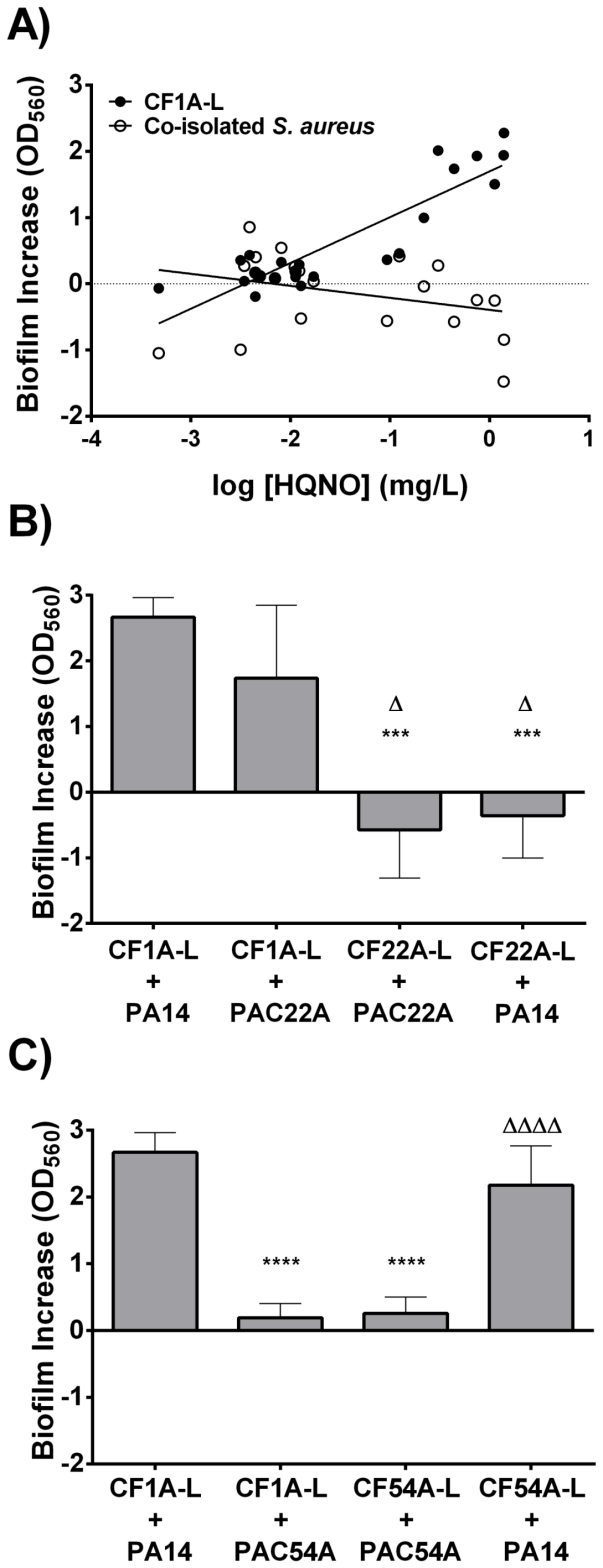
Altered response of co-isolated strains. Stimulation of *S. aureus* CF1A-L biofilm production by *P. aeruginosa* culture supernatants compared to that measured for *S. aureus* strains originally co-isolated with the studied *P. aeruginosa* isolates. (A) Correlation between HQNO production by *P. aeruginosa* strains and stimulation of *S. aureus* CF1A-L biofilm production (•) compared to that of *S. aureus* strains originally co-isolated with the studied *P. aeruginosa* isolates (○). No significant correlation is observed in the response of *S. aureus* co-isolates to the amounts of HQNO found in *P. aeruginosa* supernatants (r = -0.32, Pearson's calculation) whereas a significant correlation is seen for *S. aureus* CF1A-L (r = 0.8921; *P*<0.0001). (B) Specific example of an *S. aureus* isolate (CF22A-L) that is not stimulated for biofilm production by either PA14 or its *P. aeruginosa* co-isolate (PAC22A). There is no stimulation of biofilm production by *S. aureus* CF22A-L in comparison to that observed for *S. aureus* CF1A-L in the presence of supernatant from *P. aeruginosa* PA14 (***, *P*<0.001) or co-isolate PAC22A (Δ, *P*<0.05; one-way ANOVA and Bonferroni's multiple comparison test). (C) *P. aeruginosa* PAC54A supernatant does not increase biofilm formation of either *S. aureus* CF1A-L or its co-isolate *S. aureus* CF54A-L even though both *S. aureus* strains have their biofilm production greatly increased by the *P. aeruginosa* PA14 supernatant. Results obtained with *S. aureus* CF1A-L were used as control and significant differences between conditions are shown for both supernatants used, PA14 (****, *P*<0.0001) or PAC54A (ΔΔΔΔ, *P*<0.0001).

*S. aureus* biofilm stimulation assays were also performed using *S. aureus* isolates retrieved from patients that were co-colonized with *P. aeruginosa*. Unexpectedly, *P. aeruginosa* isolates showing the highest biofilm stimulatory activities on CF1A-L (*i.e.*, those showing the highest production of HQNO; Fig. 1C) were unable to maintain this trait toward their respective co-isolates (Fig. 4A). We found, as before, a strong correlation between biofilm stimulation of CF1A-L and HQNO levels in the *P. aeruginosa* supernatants (r = 0.8921, Pearson's correlation calculation; *P*<0.0001, two-tailed) but no correlation was found (r = -0.32) when the same supernatants were tested with their respective co-isolated *S. aureus* (Fig. 4A). In other words, the correlation between HQNO production and the stimulation of *S. aureus* biofilm production is lost when *P. aeruginosa* and *S. aureus* co-isolates are tested together.

Very interestingly, both the lack of stimulation of some *S. aureus* strains by high levels of HQNO and the lack of adequate production of HQNO by some *P. aeruginosa* strains could explain the lack of biofilm stimulatory effect among co-isolates. Fig. 4B shows a specific example of an *S. aureus* isolate (CF22A-L) that is not stimulated for biofilm production by either PA14 or its co-isolate (*P. aeruginosa* PAC22A) despite adequate production of HQNO and biofilm stimulation of the *S. aureus* reference strain CF1A-L. Similarly, 28.5% (6/21) of all the *S. aureus* co-isolates did not respond to PA14 stimulation although they were co-isolated with *P. aeruginosa* isolates causing significant stimulation of *S. aureus* CF1A-L. On the other hand, Fig. 4C shows an example of the other scenario where the supernatant from strain *P. aeruginosa* PAC54A does not increase biofilm formation of either its co-isolate *S. aureus* CF54A-L or the reference strain *S. aureus* CF1A-L while the biofilm production of both *S. aureus* strains is greatly increased by a supernatant of PA14. Indeed, the supernatant from strain *P. aeruginosa* PAC54A only contains 4% of the amount of HQNO produced in a supernatant from PA14 as determined by LC/MS. Overall, 73.9% (17/23) of the *P. aeruginosa* co-isolates produced relatively low amounts of HQNO and were poorly able to stimulate *S. aureus* CF1A-L biofilm production (as determined by Sidak's multiple comparisons test; Fig. 1C). Consequently, considering the *S. aureus* strains that do not respond to HQNO and the low-HQNO-producing *P. aeruginosa*, none of the co-isolated *S. aureus* showed biofilm augmentation when in presence of supernatants obtained by their specific *P. aeruginosa* counterparts.

All together, this suggests that some type of strain selection within the host or some type of interspecific adaptations providing either a tolerance of *S. aureus* to HQNO or a decrease production of stimulatory exoproducts by *P. aeruginosa* can occur with these bacterial species during colonization or co-colonization of CF lungs.

### Effect of co-colonization on FEV1

**Figure 5:**
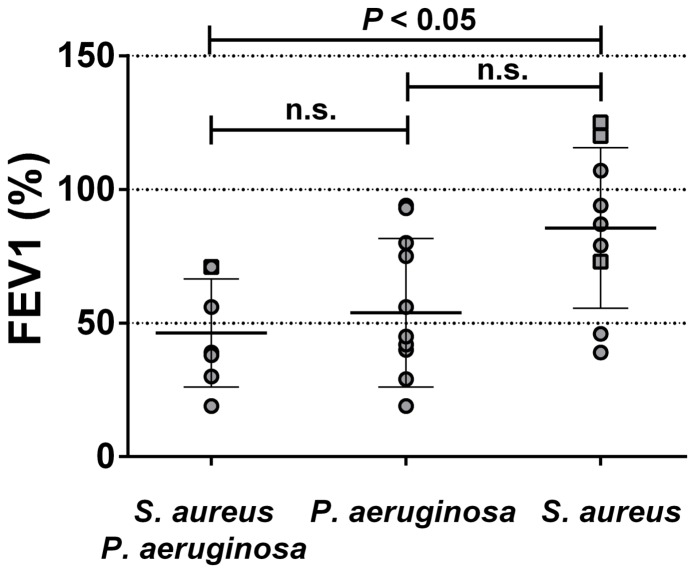
Health condition of co-colonized patients. FEV1 values in patients colonized with one or both pathogens. Each dot represents the FEV1 of a patient. Square symbols represent patient colonized by a MRSA strain. Bars show the means with standard deviations. Groups were compared using a Kruskal-Wallis test followed by Dunn's multiple comparison test (ns: non-significant).

The respiratory health, expressed by FEV1 values for each patient at the time of sampling has been compiled. FEV1 values were segregated into three groups in relation to the bacterial species colonizing the respiratory tract (Fig. 5). Although the overall number of patients for such analysis is relatively low, we note that the group of patients from which both *S. aureus* and *P. aeruginosa* were simultaneously retrieved shows the poorer breathing capacity (mean FEV1). Four patients were colonized by MRSA and only one was co-colonized with *P. aeruginosa* (Fig. 5). These results suggest that co-colonization worsens the patient health, although statistical significance was only observed between the *S. aureus* group and the *S. aureus* and *P. aeruginosa* co-colonized group.

## Discussion

We gathered a collection of 63 clinical *P. aeruginosa* isolates retrieved from the respiratory tract of 32 adult CF patients over a period of three years. From those isolates, 23 were collected from patients in whom *S. aureus* was also found co-infecting with *P. aeruginosa*. This collection thus offered a unique strain panel for investigations on the properties of *S. aureus* and *P. aeruginosa* co-isolates.

It was previously demonstrated that the *P. aeruginosa* exoproduct HQNO promotes the emergence of *S. aureus* small colony variants (SCVs) and importantly increases biofilm formation by prototypical *S. aureus* [16], [18]. Since biofilm are associated with chronic and difficult to eradicate infections [13], [25], we were therefore interested to know if a similar phenomenon could be observed with clinical isolates of both *S. aureus* and *P. aeruginosa*; in other words, to know if one species could influence some aspects of the other species colonization or pathogenesis in general.

By treating cultures of *S. aureus* strain CF1A-L with culture supernatants from the *P. aeruginosa* strain collection, we were able to demonstrate a broad range of biofilm stimulatory abilities among the clinical isolates of *P. aeruginosa*; some greatly stimulated *S. aureus* biofilm production while others did not. Noteworthy, isolates that were retrieved from same patients showed not only different morphotypes, but also different biofilm stimulatory activity on *S. aureus* CF1A-L. We subsequently used LC/MS analysis to precisely determine the types and concentrations of *P. aeruginosa* exoproducts present in culture supernatants and to identify those most likely responsible for the stimulation of *S. aureus* biofilm production. LC/MS results showed that the capacity to stimulate *S. aureus* biofilm formation was strongly associated to the production of the two most abundant AQs, HQNO and PQS. Since biosyntheses of HQNO and PQS are simultaneously derived from the same main pathway [37], [38], we used isogenic mutants deficient in some parts of the biosynthesis pathways and signaling mechanisms to further decipher the contributions of HQNO and PQS. The observation that the *pqsA* mutant, deficient in both PQS and HQNO production, demonstrates poorer biofilm stimulation than that observed using either of the *pqsH* or *pqsL* mutants deficient in PQS or HQNO production, respectively, supports some contribution of both PQS and HQNO to the *S. aureus* biofilm stimulatory activity. Besides, the fact that *pqsH* is less affected than *pqsL* in its ability to promote biofilm formation tends to indicate a greater role for HQNO. However, confounding factors may impact on such deductions. For example, the *pqsL* mutant accumulates precursors that may increase the proportions of other AQs. Also, a blocked production of PQS in the *pqsH* mutant will certainly impact on the expression of the *pqsABCDE* operon, as PQS is known to act as an inducer *in vitro*.

Since the double mutant *lasR/rhlR* is even more affected than *pqsA* in its ability to stimulate *S. aureus* biofilm formation, other factors, others than the AQs controlled by *pqsA/pqsE*, may also be involved. AHLs could represent good candidates as they were described to inhibit *agr* controlled phenotypes in *S. aureus* [7] and are regulated through the *lasR*-dependent quorum-sensing in which mutations in CF related isolates have been widely discussed [48], [49], [50]. It is also possible that other AQs from the long list of such molecules produced by *P. aeruginosa* [38] also play some role in *S. aureus* biofilm stimulation. A phenazine could also be involved but pyocyanin production did not correlate with biofilm stimulation and the *pqsA* and *pqsE* mutants produce less or no pyocyanin at all. We have also ruled out rhamnolipids (no difference between PA14 and the *rhlA* mutant, Fig. 3A). Besides, it has been reported that *P. aeruginosa* rhamnolipids could act as surfactant molecules capable of disrupting biofilms produced by Gram positive bacteria, such as *S. aureus* [51], [52]. Therefore, it would be interesting to document *S. aureus* CF1A-L apparent resilience to biofilm disruption (Fig. 3A) although we need to consider that rhamnolipids were not optimally produced in our experimental conditions (medium used and time of supernatant harvesting). This may need to be revisited in another experimental setup or with the use of purified rhamnolipids. Hence, the stimulation of *S. aureus* biofilm production by *P. aeruginosa* seems multifactorial and controlled by *lasR/rhlR*, with HQNO being one of the principal actors in this phenomenon.

To our knowledge, very few studies have examined the interactions of co-isolates of *S. aureus* and *P. aeruginosa*. Interestingly, we demonstrated here that although some *P. aeruginosa* isolates produce adequate amounts of HQNO and stimulate biofilm production of the *S. aureus* control strain CF1A-L, such *P. aeruginosa* isolates were unable to induce the same effect on the co-isolated *S. aureus* strains. Our results also demonstrated the opposite situation in which PA14 HQNO-sensitive *S. aureus* isolates were co-isolated with *P. aeruginosa* strains producing low concentrations of HQNO. Overall, these results suggest that colonization of the lungs of CF patients promotes some type of strain selection or the adaptation of either one of these pathogens to the presence of the other. These adaptations may, in one of the two scenarios, provide *S. aureus* with the ability to tolerate more efficiently the presence of pseudomonal compounds, such as HQNO that can reduce growth. In the second scenario, a deficiency of HQNO production in *P. aeruginosa* could be the result of the lung environment [48], [49], [53] or the production of 2-amino acetophenon [54], which have been reported to select for the emergence of *lasR* mutants. Indeed, absence of *lasR* results in reduced HQNO production [37]. On the other hand, the presence of *S. aureus* and its increased biofilm formation resulting from the production of HQNO may also provide some pressure on *P. aeruginosa* for selection of HQNO-deficient strains. We propose that the coexistence of *S. aureus* and *P. aeruginosa* in CF patients results from an adaptive process, notably in the interspecific interactions involving HQNO. Similarly, it has been observed that some *P. aeruginosa* genetic mutations leading to an up-regulation of siderophore production can occur in the presence of *S. aureus* in iron-limiting conditions [55]. As for the ability of *S. aureus* to adapt to the presence of *P. aeruginosa*, we suspect that the alternative SigB factor may play an important role. Indeed, we have already shown that *S. aureus* SigB can promote host colonization, emergence of SCVs and biofilm formation in response to antibiotic treatment and HQNO [18], [56], [57]. Our study supports the emerging perspective of a co-adaptation and interspecies cooperation that is largely contrasting with studies focusing on the competitive/inhibitory interactions between both bacterial species [6], [16], [17], [58], [59]. Hence, our findings are in accordance with the fact that *S. aureus* and *P. aeruginosa* are often found together in CF lungs [3], [16], [60], [61].

Studying microbial interactions and adaptive processes leading to co- or poly-bacterial infections is now evidently important. Traditional microbiology revealed *S. aureus*-positive samples in 56% of the cases in our sputum collection, whereas, PCR detection revealed *S. aureus* in as much as 77% of the samples. In contrast, *P. aeruginosa* was similarly detected in 65% and 71% of samples by microbiology and PCR, respectively. Consequently using PCR, *S. aureus* and *P. aeruginosa* co-isolate pairs were detected in 52% of cases instead of 25% (data not shown). This shows that the presence of *S. aureus* in the CF lungs of adult patients may often be underestimated and our results suggest that the co-existence of *P. aeruginosa* and *S. aureus* may potentially impact on the patient health (Fig. 5). A recent study involving many more patients observed that MRSA might be more deleterious than MSSA only when associated with *P. aeruginosa* [62]. However, it is still difficult to know if CF infections involving “adapted” *S. aureus* (or MRSA) and *P. aeruginosa* co-isolates, as opposed to prototypical “competitive” isolates, can result in a worse or better prognosis. Nevertheless, our study implies that each patient will present specific bacterial isolates with distinct properties [63]. Future patient-specific therapies may need to account for *P. aeruginosa* and *S. aureus* co-isolates' characteristics and take into consideration possible therapy-induced disturbances in the microbiota.

## Supporting Information

Figure S1 *S. aureus* CF1A-L biofilm production in response to the culture supernatant of *P. aeruginosa* PA14. Biofilm increase was measured at each supernatant concentration after 48 h of incubation and results were normalized relatively to the biofilm production measured in the absence of *P. aeruginosa* supernatant. Means and standard deviations for triplicates of each supernatant concentration are shown. Statistical significance was determined by a two-way ANOVA and the Bonferroni's multiple comparison post-test (***, *Ρ*<0.001). A supernatant from *E. coli* K12 was used as negative control.
(TIF)

Figure S2 A) *S. aureus* CF1A-L biofilm formation in function of pyocyanin production by *P. aeruginosa* isolates. Pyocyanin production by each isolate is reported relative to *P. aeruginosa* PA14's production (100%). B) CF1A-L biofilm formation in function of HHQ levels produced by *P. aeruginosa* isolates. C) CF1A-L biofilm increase in function of C4-HSL and D) 3-oxo-C12-HSL levels detected in *P. aeruginosa* supernatants. Pearson's correlations (r) are shown.
(TIF)

Table S1 Characteristics of the Pseudomonas aeruginosa clinical isolates used in this study and concentrations of exoproducts present in culture supernatants.
(XLSX)
