# Supplementary figures and images for: Interspecific Small Molecule Interactions between Clinical Isolates of Pseudomonas aeruginosa and Staphylococcus aureus from Adult Cystic Fibrosis Patients
Source: PLoS One. 2014 Jan 23;9(1):e86705. doi: 10.1371/journal.pone.0086705 (PMC3900594; doi:10.1371/journal.pone.0086705)

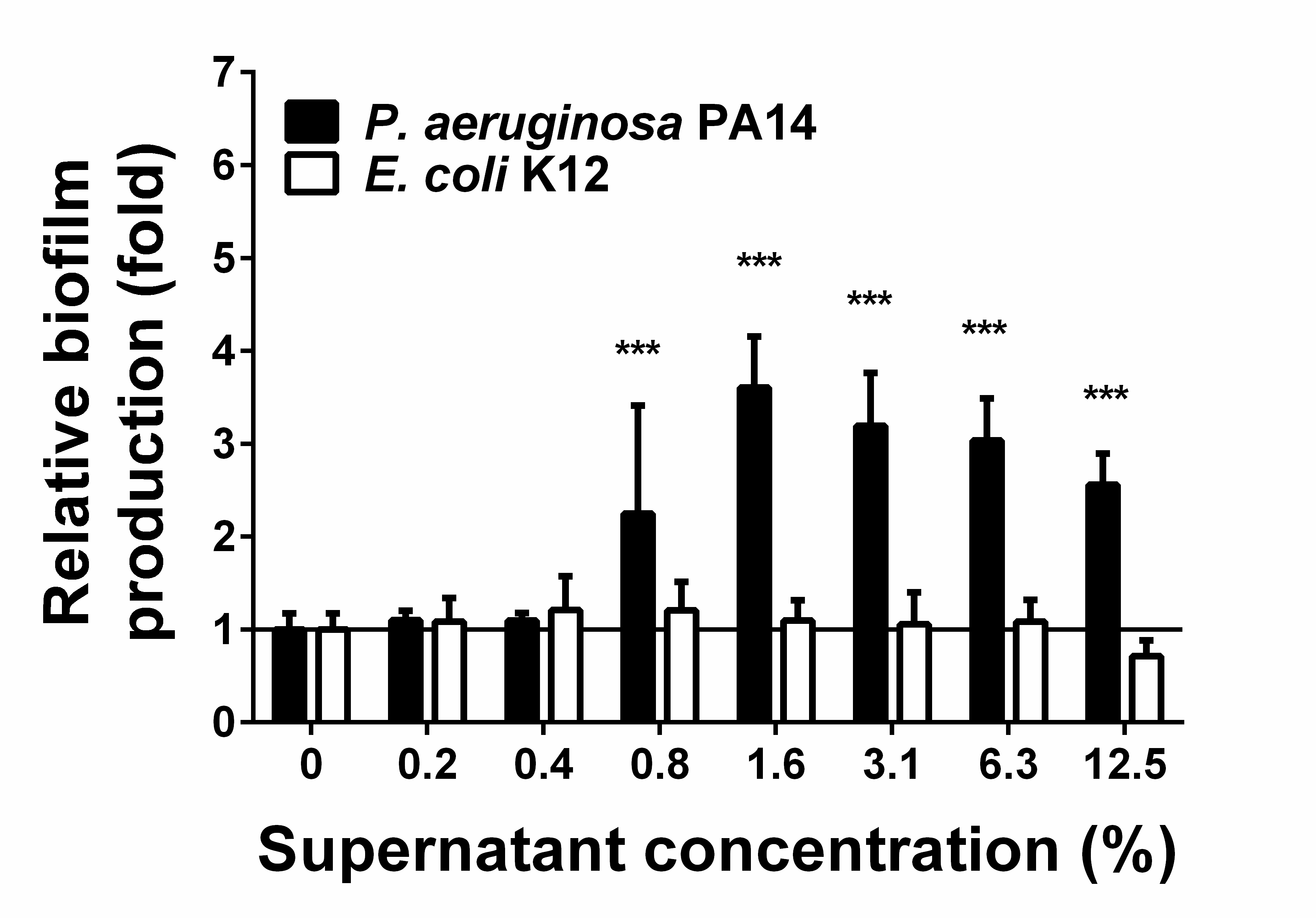

Supplement: Figure S1 — S. aureus CF1A-L biofilm production in response to the culture supernatant of P. aeruginosa PA14. Biofilm increase was measured at each supernatant concentration after 48 h of incubation and results were normalized relatively to the biofilm production measured in the absence of P. aeruginosa supernatant. Means and standard deviations for triplicates of each supernatant concentration are shown. Statistical significance was determined by a two-way ANOVA and the Bonferroni's multiple comparison post-test (***, Ρ<0.001). A supernatant from E. coli K12 was used as negative control. (TIF) [file pone.0086705.s001.tif]

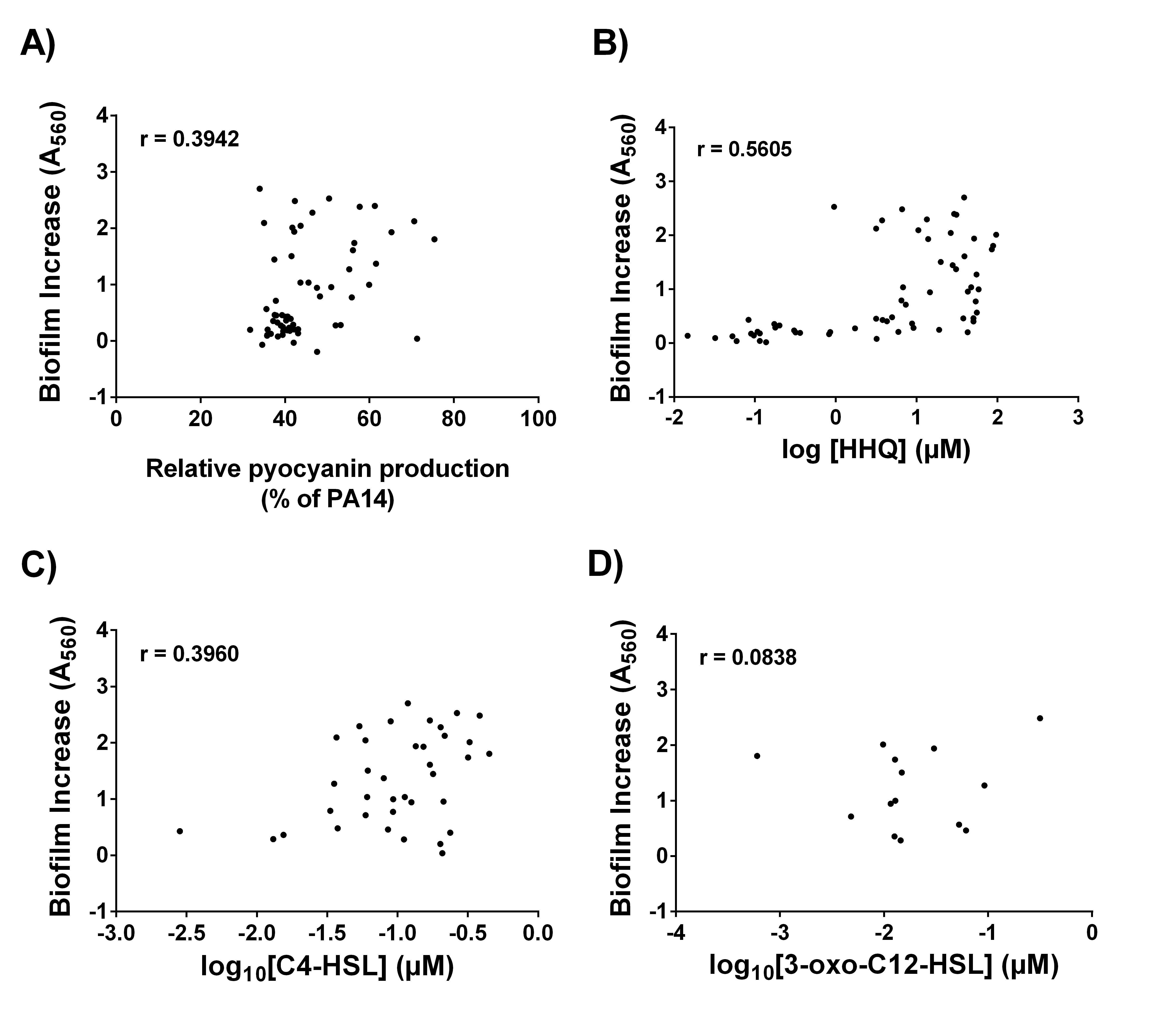

Supplement: Figure S2 — A) S. aureus CF1A-L biofilm formation in function of pyocyanin production by P. aeruginosa isolates. Pyocyanin production by each isolate is reported relative to P. aeruginosa PA14's production (100%). B) CF1A-L biofilm formation in function of HHQ levels produced by P. aeruginosa isolates. C) CF1A-L biofilm increase in function of C4-HSL and D) 3-oxo-C12-HSL levels detected in P. aeruginosa supernatants. Pearson's correlations (r) are shown. (TIF) [file pone.0086705.s002.tif]
